# Patients’ Intentions to Use Telemedicine for Ophthalmic Medical Care During the COVID-19 Pandemic

**DOI:** 10.7759/cureus.54709

**Published:** 2024-02-22

**Authors:** Michael Tsatsos, Angelos Rodafinos, Ioannis K Athanasiadis, Dafni Mavropoulou

**Affiliations:** 1 Ophthalmology, Aristotle University of Thessaloniki, Thessaloniki, GRC; 2 Ophthalmology, Modern Ophthalmic Practice, Thessaloniki, GRC; 3 Psychology and Behavioural Sciences, Aristotle University of Thessaloniki, Thessaloniki, GRC; 4 Internal Medicine, Hippokration General Hospital of Thessaloniki, Thessaloniki, GRC

**Keywords:** participants, covid-19, ophthalmology, technology acceptance, telemedicine

## Abstract

Background

This study aimed to examine the factors predicting participants’ intention to use telemedicine during the COVID-19 pandemic. Interest in health information technologies (HITs) has increased due to COVID-19. Most studies have focused on the acceptance of HIT by physicians and nurses, while there is a lack of studies on patients’ perception and acceptance of such systems in ophthalmology.

Methodology

In the first year of the COVID-19 pandemic, a survey comprising 19 items was conducted at an ophthalmic center in Greece. The participants included 77 patients diagnosed with various eye diseases. The survey aimed to evaluate variables related to the Technology Acceptance Model (TAM), such as perceived usefulness, perceived ease of use, facilitating conditions, and intention to use telemedicine. The statistical analyses included intercorrelations, internal consistency reliability tests, and multiple linear regression analysis to examine the predictors of intention to use telemedicine.

Results

The multiple linear regression analysis revealed that perceived usefulness and facilitating conditions emerged as significant predictors of eye patients’ intentions to use HIT. Interestingly, while perceived ease of use did not exhibit a significant predictive relationship with use intentions, the influence of perceived usefulness and facilitating conditions within the healthcare context underscores the pivotal role of perceived utility and external support in shaping patients’ willingness to engage with HIT for eye care.

Conclusions

Empirical data on patient acceptance offer a better understanding of the limiting factors and the variables that facilitate intentions to use services that may improve medical diagnoses, patient communication, and treatment adherence processes. Related interventions and communication efforts should highlight the benefits of HIT in ophthalmology and provide the appropriate support and means to facilitate its use during and after the pandemic.

## Introduction

Information technology is rapidly advancing, offering new medical applications that improve patient outreach, communication, and care plans. Telemedicine technologies, which have been available for over 50 years [1], have seen increased interest due to COVID-19 restrictions and lockdowns. The pandemic has significantly impacted healthcare services, causing problems for patients and enforcing dynamic changes in medical processes. Ophthalmic practice was no exception, with clinics running reduced services and postponing elective procedures [2]. Patients with non-urgent ophthalmological problems, who can use technology, prefer to stay at home and communicate with their doctors or exchange information via telecommunications technologies, benefiting from the advantages of telemedicine. Hence, many hospitals and institutions have transitioned from traditional face-to-face consultations to virtual telemedicine to reduce viral spread and comply with governmental regulations. Initially intended for primary and secondary care centers, telemedicine has been primarily used in large metropolitan areas to follow up patients with specific pathologies [3]. However, this level of telemedicine may not be feasible for populations in remote areas, with multiple pathologies, or with internet access issues.

To appreciate the usefulness of telemedicine, its diffusion and acceptance need to increase. Several models have been advocated in the past four decades to explain technology adoption. Lai [4] published a comprehensive review of the classic Theories of Reasoned Action [5], Planned Behaviour [6], and technology-related models. The latter include, among others, the Technology Acceptance Model (TAM) [7] and its extension versions TAM 2 and 3 [8,9], as well as the Unified Theory of Acceptance and Use of Technology (UTAUT) [10]. All models have been widely used over the years to explain the adoption of technology systems in various settings.

According to the review of the theories and models by Harst et al. [11] to empirically explain the acceptance of telemedicine by healthcare providers and their patients, the most commonly used predictor variables were perceived usefulness, social influences, and attitude. Healthcare providers considered the usefulness of telemedicine in their day-to-day clinical practice to be of vital importance, whereas, from a patient’s perspective, telemedicine acceptance by their social environment was very important, presumably because family and friends could help them use the technology if needed. Most studies have focused on the acceptance of physicians and nurses. There is a lack of studies on patients’ perception and acceptance of health information technologies (HITs; electronic health records, telemedicine, health information exchanges, and other digital tools that aim to improve the efficiency, accuracy, and accessibility of healthcare data and services) in ophthalmology, while there are hardly any studies conducted throughout the COVID-19 pandemic.

Even before the peak of the COVID-19 pandemic, the American Academy of Ophthalmology predicted the rise of telemedicine use in ophthalmology [12]. However, the adoption rate of telemedicine among doctors during the early months of the pandemic was not as high as expected. Tailoring training, support, and updates on regulations and billing where it is mostly needed may increase telemedicine adoption across ophthalmic services and ophthalmologists [13].

Telemedicine is expected to gradually become a more accepted diagnostic and treatment modality for chronic and acute ophthalmic diseases. According to Sommer and Blumenthal [14], both medical personnel and healthcare users consider remote care as a safer alternative to in-house visits allowing more patients to be screened in a given period. Improvements in imaging technology, artificial intelligence (AI) algorithms, and legal, financial (billing), and other bureaucratic processes may accelerate patient acceptance of telemedicine for ophthalmic medical chronic and acute services.

Teleophthalmology has been considered for years as a tool for the remote and underserved. Yet, the COVID-19 pandemic and rolling restrictions on travel and movement have made almost everyone remote and underserved. This, alongside the increasing interest from patients of all age groups, state regulations, and healthcare reimbursement, is likely to lead to a more permanent role for telemedicine in ophthalmology [15].

Empirical data on patient acceptance of telemedicine would offer a better understanding of the limiting factors and the variables that facilitate intentions to use services that may improve medical diagnoses, communication, and treatment adherence processes. Hence, this study aimed to assess the participants’ intention to use telemedicine during the COVID-19 pandemic. Thus, the purpose of the present questionnaire-based (prospective) study was to examine the factors that predict eye patients’ intentions to use information technology for health purposes during the pandemic. It was expected that participants’ attitudes and facilitating conditions would predict telemedicine usage intentions.

## Materials and methods

Participants and procedure

Questionnaires were administered to 90 adult participants with eye diseases in a single practice office in Thessaloniki, Greece. Of them, 86% (N = 77, Mage = 46.4, SD = 13.2, 61% females) returned the completed questionnaires. The study was conducted between July and October 2020, following Greece’s first lockdown (from April to June) and before the second wave of COVID-19 and lockdown in November when commuting and travel restrictions had been lifted and clinical visits returned to pre-pandemic levels resembling the “normal” clinic occupancy and population [16]. The sampling method employed was convenience sampling, where patients visiting the clinic were invited to participate. Participants completed a questionnaire either online or while waiting to be admitted for their examination. Following international guidelines for research ethics, participation was voluntary and all participants were informed about the purposes of the study and signed a consent form. Local Institutional Ethics Committee approval was obtained (approval number: 07/H0512/39), and the study adhered to the World Medical Association principles, as described in the Declaration of Helsinki.

Measures

A 19-item questionnaire was given to participants to assess their attitudes and intentions to use telemedicine. According to the original TAM model, acceptance and, subsequently, use of telemedicine is a function of perceived usefulness and perceived ease of use. Social influence and facilitating conditions are among the most common predictors added to the TAM [11]. We did not include social influences because they do not appear to have an effect in voluntary contexts [10]. Facilitating conditions include internet access, related machinery or instrumentation, and knowledge of how to use these. We included facilitators in this study because their presence has been found to be a significant predictor of telemedicine acceptance [17].

Perceived usefulness refers to making the care process more convenient for the participant which includes the following items: make my interaction with the ophthalmologist easier; help me save time from commuting to the ophthalmologist; enable access to the doctor quicker (e.g., exchange of information, documents, files); satisfy some of my diagnosis needs; help me book and remember my appointments; improve the course of my treatment/therapy (e.g., by receiving text reminders about my medication, follow-up exams); and save money.

Perceived ease of use refers to understanding and learning or knowing how to use information technology which includes the following items: it will be easy for me to learn how to use telemedicine services effectively; the use of telemedicine services will be easy and clear; and the use of telemedicine services will not require much effort on my part.

Facilitating conditions were assessed with items such as the availability of technological equipment and the use of blog/social network accounts and messaging services. Specific items included ownership of devices with internet access (smartphones, computers, tablets); knowing how to use them; experience of use of electronic services for transactions (e.g., e-banking, bill payment); familiarity with social networks to communicate with the people (e.g., Facebook, Twitter, Instagram, WhatsApp, Viber, Skype); knowing how to use telemedicine services effectively; and availability of people in close contact who can assist with telemedicine services.

Finally, participants were asked to state their intentions to use telemedicine (as defined earlier) in the next three months for the following purposes: ophthalmologist consultations via video conference (Skype, Viber, Facebook, WhatsApp); remote diagnosis on simple matters; receiving message reminders (for medical appointments, to take one’s pills, therapy); and exchange of files (images, prescriptions, exam results). Hence, intention to use telemedicine was the dependent variable, while predictor variables included perceived usefulness, perceived ease of use, and facilitating conditions.

The degree of participants’ agreement with all statements was measured using a five-point Likert scale ranging from 1 (not at all) to 5 (very much) (e.g., I believe that telemedicine will make my interaction with the ophthalmologist easier). Facilitating conditions were assessed using a binary yes/no scale.

With 77 participants and three predictors, the sample size provided sufficient power (0.80) for multiple linear regression (α = 0.05). The model’s R-squared (0.60) was higher than the minimum detectable effect size (0.40), confirming adequacy.

Statistical analysis

The statistical analyses used in this study included intercorrelations among key variables, Cronbach’s alpha values for internal consistency reliability, and multiple linear regression analysis to examine the predictors of intentions to use telemedicine. Two approaches were used to check for heteroscedasticity, namely, visual inspection and the Breusch-Pagan test. The presence of multicollinearity among predictor variables was examined using the variance inflation factor (VIF) values which quantifies the extent of correlation between predictors.

## Results

An examination of the questionnaire’s multi-item measures underscored the robust internal consistency reliability across all constructs, with Cronbach’s alpha values consistently exceeding 0.71. This robustness instills confidence in the stability and reliability of the measurement instruments, establishing a strong foundation for the analyses.

Regarding the intercorrelations among key variables, namely, perceived usefulness, perceived ease of use, facilitating conditions, and intentions to use telemedicine, we found substantial and statistically significant associations, aligning with the anticipated relationships posited by TAM. Notably, perceived usefulness exhibited a pronounced positive correlation with intentions to use telemedicine (r = 0.70, p < 0.001), underscoring its pivotal role in shaping participants’ behavioral intentions. Contrary to expectations, the absence of a significant correlation between perceived usefulness and facilitating conditions suggests a nuanced relationship. While participants recognize the utility of telemedicine, external factors may independently influence their intentions to use it. These intricate relationships are depicted in Table 1, offering a detailed overview of the interplay among these constructs.

**Table 1:** Intercorrelations, means, standard deviations, and reliability coefficients of the measures. *: p < 0.001.

	1	2	3	4
Perceived usefulness	-	0.66*	0.18	0.70*
Perceived ease of use		-	0.42*	0.63*
Facilitating conditions			-	0.45*
Intentions				-
Mean	3.94	3.69	4.96	3.53
Standard deviation	0.79	0.83	1.45	1.13
Cronbach’s alpha	0.89	0.71	0.92	0.92

A multiple linear regression analysis provided deeper insights into the determinants of intentions to use telemedicine. Overall, the model was highly significant [F (3, 74) = 37.5, p < 0.001], explaining 60% of the variance in intentions to use telemedicine (adjusted R^2^). This is a moderate multivariate effect size (F2 > 0.36) according to Cohen [18]. This substantial explanatory power, comparable to the median of UTAUT studies (R^2^ = 0.59) [11], attests to the robustness of the predictive model.

Perceived usefulness and facilitating conditions emerged as significant predictors of intentions (β = 0.57, p < 0.001 and β = -0.28, p < 0.001, respectively), while perceived ease of use did not attain significance (β = 0.14, p = 0.12). These findings, summarized in Table 2, shed light on the distinct impact of these factors on participants’ intentions to adopt telemedicine.

**Table 2:** Predictors of intentions to use telemedicine. *: p < 0.001.

Predictors	β	95% CI	Adjusted R^2^
Perceived usefulness	0.57*	0.51–1.05	
Perceived ease of use	0.14	-0.10–0.46	
Facilitating conditions	-0.28*	-2.58–-0.54	0.60

Heteroscedasticity was assessed using visual inspection and the Breusch-Pagan test. Although the visual inspection showed some minor deviations from normality, these deviations were not substantial enough to conclude the presence of heteroscedasticity. The Breusch-Pagan test also failed to reject the null hypothesis of homoscedasticity (F = 0.38, p = 0.77). These results suggest that the assumption of homoscedasticity is met, which supports the validity of the regression results. A P-P plot showed close alignment with the diagonal, suggesting the normality of residuals.

The VIF values were employed to examine the presence of multicollinearity among predictor variables. VIF values for our variables were 1.23, 1.80, and 2.10, consistently below the threshold of 10 and well within the acceptable range.

Moreover, analysis of variance unveiled a lack of gender differences in any of the model’s factors. Notably, age exhibited a negative relationship with facilitating conditions (r = -0.37, p < 0.01), suggesting that older individuals may encounter more challenges in accessing external support for utilizing telemedicine.

## Discussion

This study set out to assess a modified version of the UTAUT model among eye patients. It was hypothesized that eye participants’ intentions to use HIT systems would be predicted by core model variables, such as perceived usefulness, perceived ease of use, and facilitating conditions. Results showed that perceived usefulness and facilitating conditions, but not perceived ease of use, significantly predicted telemedicine usage intentions. The moderately strong predictive value of the two variables is in line with previous studies of technology acceptance in healthcare settings [19].

Regarding perceived usefulness, findings indicated that during the period of restricted commute and travel, participants were willing to explore alternative ways to make the interaction between themselves and their eye doctor as easy and efficient as possible. It is interesting to note that the items that ranked higher within the perceived usefulness of the telemedicine scale were “Speed of access to the doctor” and “Help me save time from commuting to the ophthalmologist.” This comes as no surprise as professional businesses and interactions with clients did not cease completely. Even in the retail industry, which was hit harder and saw shop closures, electronic trade or click-away modes were employed.

Perceived ease of use of telemedicine, on the other hand, was not a predictor of participants’ intentions to use telemedicine. Perhaps, compared to past studies, perceived ease of use has lost part of its significance in the model because people today are more proficient in using technology, as they have already overcome the steep learning curve and the initial frustration, thus, no longer feeling discouraged or considering telemedicine an obstacle in receiving quality ophthalmic care. Another reason could be that participants realized that the restrictions and rolling lockdowns were here to stay, slowly becoming a part of life’s routine. Hence, they had to find and invest the required time to experiment with telemedicine in exchange for safe and timely access to ophthalmic care.

Facilitating conditions, such as the ownership of smartphones or similar devices and previous experience with social networks, was a significant predictor of intentions to use telemedicine. During the first wave of the pandemic, the newly elected government introduced several measures and incentives to reduce bureaucracy and speed up processes such as remote health appointments, electronically issued prescriptions for high-cost medications (e.g., intravitreal injections), and subsidy of spectacle prescriptions. These probably increased participant awareness of the potential and benefits of the use of technology in telemedicine. Although these measures and incentives were introduced only recently, they seem to have high diffusion in society, appear to have had a positive impact, and could be used as a guide for similar future initiatives.

In terms of intentions, although participants of all age groups appeared to be relatively open when it came to telemedicine use for certain services, e.g., to allow speedy access to their ophthalmologist, they were somewhat reluctant to use the full spectrum of telemedicine opportunities. They reported that they intend to use telemedicine mainly for the exchange of files (e.g., images, prescriptions, exam results) and receiving message reminders (e.g., for medical appointments, to take one’s pills, therapy), and less for remote examination and diagnosis, if possible, or for ophthalmologist consultations via video conference (Skype, Viber, Facebook, WhatsApp). This indicates that patients are not ready to replace their physical visits to the ophthalmologist with telemedicine, probably rightfully so, as diagnostic services have not been fully developed yet and everyone involved would probably benefit from training and education on all available resources.

The COVID-19 pandemic and its accompanying restrictions on travel, gatherings in communal areas, and close interpersonal contact such as detailed face-to-face medical examinations have created a significant backlog of clinical appointments and elective surgery. Furthermore, even after the relaxation of lockdown restrictions and the restoration of some semblance of normality, a significant number of patients with multiple comorbidities experienced fear or were explicitly prohibited from traveling for their routine, scheduled, or even urgent medical appointments. As Ting et al. [19] stated, “In view of the significant backlog of work and ongoing social distancing measures, adoption of telemedicine in ophthalmology will likely become the new ‘norm’ of ophthalmology during and after the pandemic.”

The limitations of our study include using a single section of eye patients attending a private practice center in a large urban Greek city in northern Greece. The small sample size from a single clinic limits the generalizability of findings. Future studies should examine larger samples of older, rural, or remote populations (who are most likely to benefit from telemedicine), along with other populations. Another limitation is that most related studies, including ours, use self-report methodologies and focus on perceptions and not actual behaviour regarding telemedicine [20] (for an extensive discussion of related issues in this line of research see [21]). Finally, using convenience sampling possibly introduced bias.

## Conclusions

As the world emerges from the recent pandemic, understanding barriers to technology adoption becomes pivotal for devising effective strategies and guiding healthcare service implementation. This study aimed to assess the factors that predict eye patients’ intentions to use telemedicine during the COVID-19 pandemic. Results showed that perceived usefulness and facilitating conditions significantly predicted telemedicine usage intentions, while perceived ease of use did not attain significance. The study underscores the utility of TAMs in comprehending patient perspectives and beliefs and highlights an opportune moment for healthcare professionals to endorse and promote the use of existing and novel information technology applications, positively impacting treatment and quality of life. Future research should explore whether these findings extend to people of varying socioeconomic status utilizing public healthcare facilities, as well as assess potential negative consequences alongside the benefits of telemedicine.
